# Cell Division by Longitudinal Scission in the Insect Endosymbiont *Spiroplasma poulsonii*

**DOI:** 10.1128/mBio.00881-16

**Published:** 2016-07-26

**Authors:** Elodie Ramond, Catherine Maclachlan, Stéphanie Clerc-Rosset, Graham W. Knott, Bruno Lemaitre

**Affiliations:** a Global Health Institute, School of Life Science, École Polytechnique Fédérale de Lausanne (EPFL), Lausanne, Switzerland; b Interdisciplinary Centre for Electron Microscopy, École Polytechnique Fédérale de Lausanne (EPFL), Lausanne, Switzerland

## Abstract

*Spiroplasma* bacteria are highly motile bacteria with no cell wall and a helical morphology. This clade includes many vertically transmitted insect endosymbionts, including *Spiroplasma poulsonii*, a natural endosymbiont of *Drosophila melanogaster*. *S. poulsonii* bacteria are mainly found in the hemolymph of infected female flies and exhibit efficient vertical transmission from mother to offspring. As is the case for many facultative endosymbionts, *S. poulsonii* can manipulate the reproduction of its host; in particular, *S. poulsonii* induces male killing in *Drosophila melanogaster*. Here, we analyze the morphology of *S. poulsonii* obtained from the hemolymph of infected *Drosophila*. This endosymbiont was not only found as long helical filaments, as previously described, but was also found in a Y-shaped form. The use of electron microscopy, immunogold staining of the FtsZ protein, and antibiotic treatment unambiguously linked the Y shape of *S. poulsonii* to cell division. Observation of the Y shape in another *Spiroplasma*, *S. citri*, and anecdotic observations from the literature suggest that cell division by longitudinal scission might be prevalent in the *Spiroplasma* clade. Our study is the first to report the Y-shape mode of cell division in an endosymbiotic bacterium and adds *Spiroplasma* to the so far limited group of bacteria known to utilize this cell division mode.

## IMPORTANCE

Most bacteria rely on binary fission, which involves elongation of the bacteria and DNA replication, followed by splitting into two parts. Examples of bacteria with a Y-shape longitudinal scission remain scarce. Here, we report that *Spiroplasma poulsonii*, an endosymbiotic bacterium living inside the fruit fly *Drosophila melanogaster*, divide with the longitudinal mode of cell division. Observations of the Y shape in another *Spiroplasma*, *S. citri*, suggest that this mode of scission might be prevalent in the *Spiroplasma* clade. *Spiroplasma* bacteria are wall-less bacteria with a distinctive helical shape, and these bacteria are always associated with arthropods, notably insects. Our study raises the hypothesis that this mode of cell division by longitudinal scission could be linked to the symbiotic mode of life of these bacteria.

## Observation

*Spiroplasma* bacteria are members of the *Mollicutes* class, a wall-less eubacterial group related to Gram-positive bacteria (1). These bacteria exhibit a distinctive helical shape and high motility. *Spiroplasma* bacteria are widely associated with arthropods and found in 5 to 10% of all insect species. While some species are horizontally transmitted insect pathogens or commensals in the gut, other lineages exhibit transovarial vertical transmission from mother to offspring. *Spiroplasma poulsonii* is one of the species exhibiting vertical transmission, and together with *Wolbachia*, are two natural endosymbionts known from *Drosophila melanogaster.*

**FIG 1:**
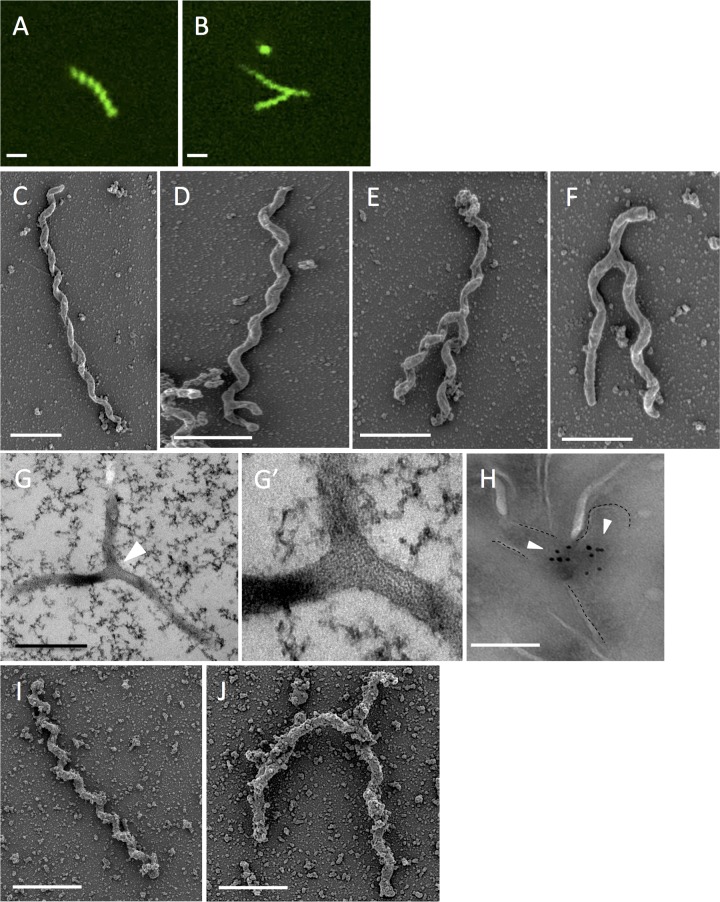
Presence of Y-shaped *S. poulsonii* in *Drosophila* hemolymph samples. (A and B) Fluorescence microscopy images showing SYTO9-stained *S. poulsonii* from freshly extracted hemolymph from 1-week-old *Drosophila melanogaster* flies. Bars, 1 µm. (C to F) SEM of *S. poulsonii* extracted from 1-week-old *Spiroplasma*-infected female flies. *S. poulsonii* can be found as one elongated body (C) or with a Y-shape conformation (D to F) with variation in the length of the arms. Bars, 1 µm. (G and zoom in G′) TEM of *S. poulsonii* from freshly extracted *Drosophila* hemolymph. The white arrowhead shows the branching. Bar, 1 µm. (H) Immunogold labeling pattern of the FtsZ protein with an anti-FtsZ antibody. Y-shaped bacteria have two FtsZ protein aggregates. Bar, 200 nm. (I and J) SEM of *in vitro*-cultured *S. citri*. *S. citri* can be found as one elongated body (I) or with a Y-shaped conformation (J). Bars, 1 µm. The presence of aggregates might be due to protein enrichment in the medium used to cultivate *S. citri*.

*S. poulsonii* bacteria are primarily found in the hemolymph of adult flies, where they are neither detected nor affected by the *Drosophila* immune system. The proliferation of *Spiroplasma* is constrained by the availability of hemolymph lipids (2, 3). *S. poulsonii* uses the yolk uptake machinery to colonize the germ line during oogenesis to ensure vertical transmission (4). Similar to other facultative endosymbionts, *S. poulsonii* can affect the sex ratio of its host by inducing male killing (causing death of male embryos at early time points) (5). It has been proposed that reproductive manipulation and the ability of *Spiroplasma* to protect *Drosophila* against certain parasites are among the main driving forces that maintain this facultative endosymbiont in fly populations (6). There have been a number of studies recently investigating how *Spiroplasma* impacts its insect host; however, little is known about the biology of *S. poulsonii* itself. This led us to investigate the morphology of *S. poulsonii* (strain MSRO; for experimental methods, see Text S1 in the supplemental material) derived from fly hemolymph extracts. We chose 1- to 2-week-old females, since this time period corresponds to the exponential growth phase of this endosymbiont (2). Use of the nucleic acid stain SYTO9 reveals the presence of *Spiroplasma* with a classical helical and linear morphology (Fig. 1A). Surprisingly, up to 30% of bacteria show a Y-shape morphology, which has not been previously characterized (Fig. 1B). Scanning electron microscopy (SEM) of hemolymph samples from *Spiroplasma*-infected *D. melanogaster* females also confirms the presence of Y-shaped *Spiroplasma*. Typical linear bacteria have 5 to 15 helices, with the length of the bacteria ranging from 0.8 µm to 5 µm and the width ranging from 50 to 100 nm (Fig. 1C). SEM confirmed the presence of Y-shaped bacteria with one main branch of various sizes, splitting into two thinner arms of identical length (Fig. 1D, E, and F). To exclude the possibility that the Y-shaped form results from the twisting of two bacteria, we performed high-pressure freezing followed by transmission electron microscopy (TEM) on hemolymph samples. Our results confirm that the Y shapes correspond to one bacterium with cytoplasmic continuity between the two arms and the main trunk (Fig. 1G, white arrowhead; high-magnification view shown in Fig. 1G′).

Cytokinesis is the process by which the cytoplasm of one cell is divided in two cells. In bacteria, it involves the guanosine triphosphatase (GTPase) FtsZ (filamenting temperature-sensitive Z), which assembles at the inner face of the bacterial membrane to form a Z-ring, which subsequently recruits molecules to provide the contractile force for membrane invagination (7, 8). One gene encoding a FtsZ protein is present in the *S. poulsonii* genome (9). To assess whether the *S. poulsonii* Y shape is indeed linked to cell division, we performed immunogold labeling against FtsZ protein using a commercially available antibody. Figure 1H shows accumulation of signals corresponding to FtsZ in the branching area of Y-shape bacteria, with gold particles forming two small aggregates at the beginning of the two arms (white arrowheads). No gold particle aggregate was observed in linear bacteria.

**FIG 2:**
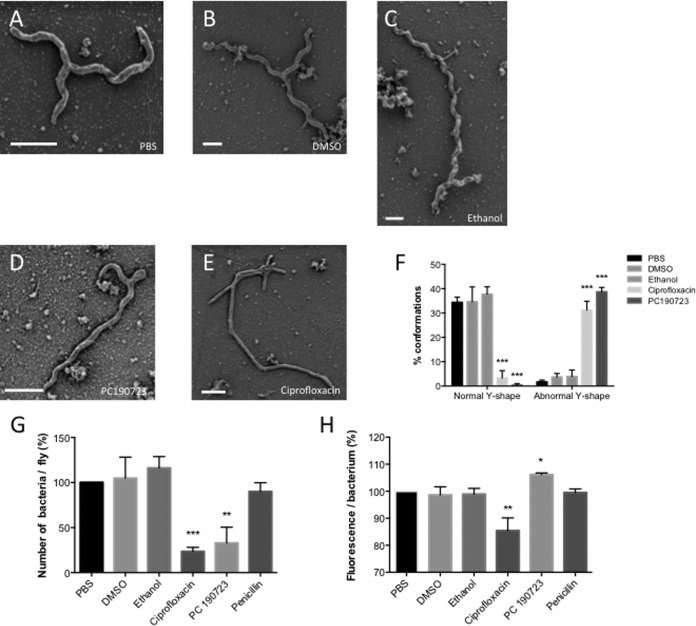
Impacts of various antibiotics on the Y-shaped form of *Spiroplasma*. (A to E) SEM of *S. poulsonii* extracted from *Spiroplasma*-infected female flies injected with phosphate-buffered saline (PBS) (A), DMSO (B), ethanol (C), PC190723 (D), or ciprofloxacin (E). One-week-old females were injected with molecules, and hemolymph was extracted after 4 days. Bars, 500 nm. (F) Quantification of normal and abnormal Y-shape *S. poulsonii* by SEM in hemolymph samples collected from treated flies. ***, *P* < 0.005. Values are means plus standard deviations (SD) (error bars) of data pooled from three independent experiments with 100 bacteria for each count. Twenty flies were used to extract fresh hemolymph for each experiment. (G) Evaluation of bacterial count per fly following each treatment. *Spiroplasma* bacteria were counted 1 week after injection. **, *P* < 0.01; ***, *P* < 0.005. Values are means plus SD of data pooled from three independent experiments with 20 flies for each count. (H) Quantification of the amount of fluorescence per bacteria. DNA fluorescence per *Spiroplasma* was measured 4 days after injection. *, *P* < 0.05; **, *P* < 0.01. Values are means plus SD of data pooled from three independent experiments with 20 flies tested for each count.

To confirm our conclusion, we analyzed the impacts of antibiotics that affect cell division on *S. poulsonii* morphology. Antibiotics were directly injected into the body cavities of *Spiroplasma*-infected flies, and hemolymph samples were collected 4 days later for observations and 1 week later for bacterial quantification in flies. Injection of PC190723 (dissolved in dimethyl sulfoxide [DMSO]), a cell division inhibitor known to block the GTPase activity of FtsZ leads to aberrant Y-shape morphology with an aborted arm situated at one extremity of the main trunk (Fig. 2D; quantified in Fig. 2F). Injection of ciprofloxacin (dissolved in ethanol), a fluoroquinolone antibiotic which inhibits DNA gyrase activity, and therefore DNA replication, results in abnormal shaped *Spiroplasma* with no spiral and multibranching with 3 to 5 arms of various sizes linked to the main trunk (Fig. 2E; quantified in Fig. 2F). We speculate that this morphology results from multiple scission departures that aborted precociously. This also suggests that DNA replication is not mandatory for the initiation of branching. Injection of DMSO or ethanol, the two antibiotic solvents, or penicillin, an antibiotic that targets peptidoglycan, a cell wall component absent in *Spiroplasma*, did not affect *S. poulsonii* morphology, indicating that the effects are caused by the two molecules (Fig. 2A to C). Figure 2G shows that PC190723 and ciprofloxacin antibiotic that affect *Spiroplasma* morphology also reduce bacterial growth as measured by quantitative PCR (qPCR).

SYTO9 DNA staining demonstrates the presence of DNA all along the arms of both linear and Y-shaped *Spiroplasma* (Fig. 1A and B). To investigate the link between DNA synthesis and *Spiroplasma* morphology, we analyze by qPCR the presence of DNA in bacteria treated with PC190723 and ciprofloxacin. Remarkably, PC190723-treated bacteria presented more fluorescence, which is consistent with the notion that FtsZ target drug inhibits cell division, but not DNA replication. In contrast, a lower DNA signal was observed upon treatment with the DNA synthesis inhibitor ciprofloxacin. Taken together, our data suggest that the production of a new arm occurs before replication of DNA but that the final scission of the two cells required DNA synthesis completion. Unfortunately, due to the high motility of *S. poulsonii* and their quick adherence (within minutes) to the substratum, we were unable to perform live imaging of *S. poulsonii* division.

Most bacteria rely on binary fission, which involved elongation of the bacteria and DNA replication before splitting into two parts. Examples of bacteria with a Y-shape division remain scarce. Bacteria with Y-shape division include *Actinomyces* (10) and *Bifidobacterium* (11). It is interesting to connect our observation to a recent study revealing the presence of the Y shape in another symbiotic bacterium. Leisch et al. have shown that the gammaproteobacterium ectosymbiont, which attaches to the surface of the marine nematode *Laxus oneistus*, grows laterally and divides longitudinally (13). This mode of cell division is thought to be important for maintaining the attachment of both daughter cells to the nematode surface (12). In contrast to *Spiroplasma*, longitudinal scission takes place simultaneously on both extremities of the ectosymbiont. Y-shaped bacteria have also been observed in another symbiotic context that involves nitrogen-fixing *Rhizobium* and leguminous plants. Y-shaped *Rhizobium* bacteria are abundant in the nitrogen fixation zone of nodules, suggesting that this morphology could be induced by the plant host (14).

Our report adds *S. poulsonii* to the short list of bacteria with a longitudinal mode of cell division and is the first to show this feature in an insect endosymbiont. Screening of the literature reveals previous observations of branched morphologies in tick-associated *Spiroplasma ixodetis* (15), plant-associated *Spiroplasma citri* (16), and an unidentified *Spiroplasma* subspecies found in the Chinese mitten crab *Eriocheir sinensis* (17). However, Garnier et al. proposed that *Spiroplasma citri* divides by elongating before constricting to give two daughter cells (18). Careful analysis of *S. citri* cultures reveals the presence of the Y shape (Fig. 1I and J), suggesting that this mode of division could be rather prevalent in the *Spiroplasma* clade. An open question is to determine the nature of the selective pressure that leads to this mode of cell division. The prevalence of the Y shape in bacteria establishing symbiotic interactions with eukaryotes may allow the coupling of longitudinal fission with signals from the host, as proposed for *Rhizobium* or the nematode ectosymbionts. Alternatively, this mode of septation might be related to the motility of *Spiroplasma*, which utilizes long contractile fibers that run from one end of the cell to the other (19). Indeed, we can speculate that division begins at one extremity of the bacteria, following by longitudinal opening (“zip-progression”) of the cell, which could explain the presence of a main trunk and two arms of the same length, before the separation of the two daughter cells on the other extremity. A longitudinal scission would allow the preservation of the fiber network such that fibers are not cut in half. Future studies should search for the presence of other Y-shaped bacteria in other microbial symbioses and characterize their functional relevance.

## SUPPLEMENTAL MATERIAL

Text S1  Experimental methods. Download Text S1, PDF file, 0.1 MB

## References

[1] Whitcomb RF 1980 The genus *Spiroplasma*. Annu Rev Microbiol 34:677–709. doi:10.1146/annurev.mi.34.100180.003333. 7002035

[2] Herren JK, Paredes JC, Schüpfer F, Arafah K, Bulet P, Lemaitre B 2014 Insect endosymbiont proliferation is limited by lipid availability. Elife 3:e02964. doi:10.7554/eLife.02964. 25027439 PMC4123717

[3] Herren JK, Lemaitre B 2011 *Spiroplasma* and host immunity: activation of humoral immune responses increases endosymbiont load and susceptibility to certain Gram-negative bacterial pathogens in *Drosophila melanogaster*. Cell Microbiol 13:1385–1396. doi:10.1111/j.1462-5822.2011.01627. 21740495

[4] Herren JK, Paredes JC, Schüpfer F, Lemaitre B 2013 Vertical transmission of a *Drosophila* endosymbiont via cooption of the yolk transport and internalization machinery. mBio 4:e00532-12. doi:10.1128/mBio.00532-12. 23462112 PMC3585447

[5] Williamson DL, Sakaguchi B, Hackett KJ, Whitcomb RF, Tully JG, Carle P, Bové JM, Adams JR, Konai M, Henegar RB 1999 *Spiroplasma poulsonii* sp. nov., a new species associated with male-lethality in *Drosophila willistoni*, a neotropical species of fruit fly. Int J Syst Bacteriol 49:611–618. doi:10.1099/00207713-49-2-611. 10319483

[6] Xie J, Tiner B, Vilchez I, Mateos M 2011 Effect of the *Drosophila* endosymbiont *Spiroplasma* on parasitoid wasp development and on the reproductive fitness of wasp-attacked fly survivors. Evol Ecol 53:1065–1079. doi:10.1007/s10682-010-9453-7. 22912533 PMC3422375

[7] Adams DW, Errington J 2009 Bacterial cell division: assembly, maintenance and disassembly of the Z ring. Nat Rev Microbiol 7:642–653. doi:10.1038/nrmicro2198. 19680248

[8] Lutkenhaus J, Pichoff S, Du S 2012 Bacterial cytokinesis: from Z ring to divisome. Cytoskeleton 69:778–790. doi:10.1002/cm.21054. 22888013 PMC3931253

[9] Paredes JC, Herren JK, Schüpfer F, Marin R, Claverol S, Kuo C-H, Lemaitre B, Béven L 2015 Genome sequence of the *Drosophila melanogaster* male-killing *Spiroplasma* strain MSRO endosymbiont. mBio 6:e02437-14. doi:10.1128/mBio.02437-14. 25827421 PMC4453565

[10] Bowden GHW 1996 *Actinomyces*, *Propionibacterium propionicus*, and *Streptomyces*. *In* Baron S (ed), Medical microbiology, 4th ed. University of Texas Medical Branch, Galveston, TX. 21413327

[11] Husain I, Poupard JA, Norris RF 1972 Influence of nutrition on the morphology of a strain of *Bifidobacterium bifidum*. J Bacteriol 111:841–844. 5053884 10.1128/jb.111.3.841-844.1972 PMC251364

[12] Polz MF, Felbeck H, Novak R, Nebelsick M, Ott JA 1992 Chemoautotrophic, sulfur-oxidizing symbiotic bacteria on marine nematodes: morphological and biochemical characterization. Microb Ecol 24:313–329. doi:10.1007/BF00167789. 24193210

[13] Leisch N, Verheul J, Heindl NR, Gruber-Vodicka HR, Pende N, den Blaauwen T, Bulgheresi S 2012 Growth in width and FtsZ ring longitudinal positioning in a gammaproteobacterial symbiont. Curr Biol 22:R831–R832. doi:10.1016/j.cub.2012.08.033. 23058799

[14] Kondorosi E, Mergaert P, Kereszt A 2013 A paradigm for endosymbiotic life: cell differentiation of *Rhizobium* bacteria provoked by host plant factors. Annu Rev Microbiol 67:611–628. doi:10.1146/annurev-micro-092412-155630. 24024639

[15] Tully JG, Rose DL, Yunker CE, Carle P, Bové JM, Williamson DL, Whitcomb RF 1995 *Spiroplasma ixodetis* sp. nov., a new species from *Ixodes pacificus* ticks collected in Oregon. Int J Syst Bacteriol 45:23–28. doi:10.1099/00207713-45-1-23. 7857803

[16] Fudl-Allah AE-SA, Calavan EC 1974 Cellular morphology and reproduction of the mycoplasmalike organism associated with citrus stubborn disease. Phytopathology 64:1309–1313.

[17] Wang W, Chen J, Du K, Xu Z 2004 Morphology of *Spiroplasmas* in the Chinese mitten crab *Eriocheir sinensis* associated with tremor disease. Res Microbiol 155:630–635. doi:10.1016/j.resmic.2004.04.010. 15380550

[18] Garnier M, Clerc M, Bové JM 1984 Growth and division of *Spiroplasma citri*: elongation of elementary helices. J Bacteriol 158:23–28. 6715280 10.1128/jb.158.1.23-28.1984 PMC215373

[19] Shaevitz JW, Lee JY, Fletcher DA 2005 *Spiroplasma* swim by a processive change in body helicity. Cell 122:941–945. doi:10.1016/j.cell.2005.07.004. 16179261

