# Supplementary material for: Cell Division by Longitudinal Scission in the Insect Endosymbiont Spiroplasma poulsonii
Source: mBio. 2016 Jul 26;7(4):e00881-16. doi: 10.1128/mBio.00881-16 (PMC4981714; doi:10.1128/mBio.00881-16)
Supplement: Text S1 — Experimental methods. Download [file mbo004162916s1.pdf]

Material and methods

Fly stocks and *S. poulsonii* extraction

We used wild-type *Oregon-R* (*OR^R*) fly stock harboring MSRO *Spiroplasma*, a strain which naturally infects *D. melanogaster,* (20). As *Spiroplasma poulsonii* is temperature sensitive (21), all flies were maintained at 25°C. As *Spiroplasma poulsonii* are still uncultivable, all experiments were performed on freshly extracted *Spiroplasma poulsonii* hemolymph. Pure *Drosophila* hemolymph was collected from flies individually using a Drummond^{TM} Nanoject and pulled in capillary needle (9). 20 to 25 flies were used per sample. *S. citri* was cultivated as described (22).

Antibiotic injection experiments

20 one-week-old *Spiroplasma* infected females were injected in lateral thorax with 9.6nl of different solutions using a Drummond^{TM} Nanoject: PBS, DMSO, ethanol, PC190723 (50mM, Merck Millipore), ciprofloxacin (20µg/ml, Sigma Aldrich, St. Louis, MO, USA) or penicillin (2.10^5 U/ml, Sigma Aldrich, St. Louis, MO, USA). Electron microscopy observations, and evaluation of fluorescence per baterium were performed 4 days and one week after injection. Evaluation of bacterial loading was performed 1 week after injection.

Examination of Spiroplasma conformation by fluorescence microscope and quantification

To observe *Spiroplasma* in fly hemolymph, flies were dissected on microscope slides in 5 µl PBS containing 0.02 mM SYTO9 (Invitrogen, Carlsbad, CA, USA). Slides were then mounted and observed on an Axioimager Z1 (Zeiss, Oberkochen, Germany). Images were captured with an Axiocam MRn camera and Axiovision software. To determine the absolute number of bacteria per fly, we extracted hemolymph from 20 infected flies homogenized in 20 µl of PBS. We performed 4 serial dilutions followed by a SYTO9 treatment. *Spiroplasma* cell numbers were counted by fluorescence microcopy as described above. To estimate the amount of DNA per bacterium, the same diluted hemolymph extracts were suspended in 20 µl of water, heated at 95°C for 20 min to release DNA in buffer and

centrifuged at 13,000 rpm for 20 min to remove debris. *DNA* was then determined using a Roche Lightcycler (LC) 2.0 detection system. For each 10 µl qPCR reaction, 5 µl of supernatant was mixed with 5 µl of LightCycler® 480 SYBR Green I Master mix according manufacturer instructions (Roche). Spiroplasma dnaA gene primers used were DnaA109F 5’-TTAAGAGCAGTTTCAAAATCGGG-3′ and DnaA246R 5′-TGAAAAAAACAAACAAATTGTTATTACTTC-3′. The amount of DNA per bacterium was obtained by the ration between the quantity of DNA as measured by qPCR and the number of bacteria.

Scanning electron microscopy (SEM)

Drops of cells contained in 50µl of PBS for *S. poulsonii* extracted from hemolymph, or 50µl of SP4 medium for *S. citri* were spotted onto a silicon wafer. These were left for 15 minutes before being fixed for 1 hour with 1.25% glutaraldehyde and 1% tannic acid in 0.1 M phosphate buffer, pH 7.4. The wafers were then washed in phosphate buffered saline, fixed again in 1.0 % osmium tetroxide in the same buffer for 30 minutes, and then dehydrated in a graded alcohol series, being drying by passing them through the critical point of carbon dioxide (Leica Microsystems CPD300). The samples were then coated with a 2nm layer of osmium using an osmium plasma coater (Filgen OPC60).

Scanning electron microscopy images were taken with a field emission scanning electron microscope (Merlin, Zeiss NTS, Germany) using an acceleration voltage of 2kV and the in-lens secondary electron detector.

Transmission electron microscopy (TEM)

Cells were preserved for transmission electron microscopy using high pressure freezing and freeze substitution. This required loading the cells in the 3 mm aluminium carrier and freezing them with a high-pressure freezer (Leica HPM100, Leica Microsystems). These samples were then placed into tubes, held in liquid nitrogen, containing frozen acetone with 1 % osmium tetroxide, 0.5% uranyl acetate, and 5% water. The tubes were then left overnight inside an ice bucket containing dry ice, agitating gently on a shaker table. The following day the dry ice was removed and the ice bucket and sample tubes allowed to warm to 0°C over the next 2 hours. At this point the acetone was replaced, three times, with fresh acetone at 5 ° C, and then

embedded in increasing concentrations of epon resin, and then placed into molds and cured for 48 hours at 65° C.

Thin (50 nm) sections of the embedded cells were cut using a diamond knife and ultramicrotome (Leica UC7). Sections were collected on single slot copper grids with a formvar support film, and then stained with lead citrate and uranyl acetate. These were imaged inside a transmission electron microscope at 80 kV (Tecnai Spirit, FEI Company), using a CCD camera (Eagle, FEI Company).

Cryo immuno electron microscopy

For immuno-electron microscopy, cells were chemically fixed with a buffered mix of 2% paraformaldehyde and 0.1% glutaraldehyde, and embedded in 12% gelatin. Small cubes (1 mm width) were then cut with a razor blade and then infiltrated overnight in 2.3M sucrose before freezing in liquid nitrogen. These were then sectioned at 100nm using an ultramicrotome (Leica UC7/FCS, Leica Microsystems, Vienna) set at 100°C. Sections were collected on formvar coated grids carrying a drop of methylcellulose/sucrose at room temperature and then processed for immunogold labeling with polyclonal antibody anti-FtsZ (rabbit, Agrisera).

Figure Legends

Figure 1. Presence of Y-shaped *S. poulsonii* in *Drosophila* hemolymph samples

(A, B) Fluorescent microscopy images showing SYTO-9 stained *S. poulsonii* from freshly extracted *Drosophila* hemolymph of 1 week-old flies. Bars, 1 µm. (**C-F)** SEM of *S. poulsonii* extracted from 1 week-old *Spiroplasma* infected females. *S. poulsonii* can be found as one elongated body (C) or with a Y-shape conformation (D-F) with variation in arms length. Bars, 1µm. (**G)** TEM microscopy of *S. poulsonii* from freshly extracted *Drosophila* hemolymph. White arrowhead shows branching. Bar, 1 µm. (**H)** Immunogold labeling pattern of FtsZ protein with an anti-FtsZ antibody. Y-shaped bacteria have two FtsZ protein aggregates. Bar, 200nm. (**I, J)** SEM microscopy of *in vitro* cultured *S. citri*. *S. citri* can be found as one elongated body (I) or with an Y-shaped conformation (J). Bars, 1 µm. The presence of aggregates might be due to proteins enrichment in the medium used to cultivate *S. citri*.

Figure 2. Impacts of various antibiotics on Y-shaped form Spiroplasma

(A-E) SEM of *S. poulsonii* extracted from *Spiroplasma* infected females injected with PBS (A), DMSO (B), ethanol (C), PC190723 (D) or ciprofloxacin (E). One-week-old females were injected with molecules, and hemolymph was extracted after 4 days. Bars, 500nm. **(F)** Quantification of normal and abnormal Y-shape *S. poulsonii* by SEM in hemolymph samples collected from treated flies. p=0.05. Shown is the mean ± SD of data pooled from three independent experiments with 100 bacteria for each count. 20 flies were used to extract fresh hemolymph for each experiment. (**G)** Evaluation of bacterial count per fly following each treatment. *Spiroplasma* were counted one week after injection. p=0.05. Shown is the mean ± SD of data pooled from three independent experiments with 20 flies for each count. (**H)** Quantification of fluorescence amount per bacteria. DNA fluorescence per *Spiroplasma* was measured 4 days after injection. *p*=0.05. Shown is the mean ± SD of data pooled from three independent experiments with 20 flies tested for each count.
